# Cauda equina syndrome in a patient with human immunodeficiency virus and secondary central nervous system lymphoma: a case report

**DOI:** 10.1186/s13256-023-04212-5

**Published:** 2023-11-15

**Authors:** Alexander Tang, David Di Fonzo, Mohammed Redha, Michael Churchill-Smith

**Affiliations:** Division of General Internal Medicine, McGill University Health Centre, Montreal, Canada

**Keywords:** Immunocompromised, Lymphoma, HIV, Cauda equina syndrome

## Abstract

**Background:**

Secondary central nervous system lymphoma (SCNSL) is a known complication of immunocompromised patients with most cases involving the brain parenchyma. Reports of cauda equina syndrome (CES) caused by SCNSL are exceedingly scarce as involvement of this anatomical region is extremely uncommon.

**Case presentation:**

We report a case of a 46-years-old, African, female patient with human immunodeficiency virus (HIV) who developed CES in the context of SCNSL. There were no blasts present in the peripheral blood smear. We provide a review of the literature, discussion of the clinical evolution of this patient and the radiological/histopathological findings. The patient ultimately responded well to induction chemotherapy and high dose methotrexate.

**Conclusion:**

This case report demonstrates that CES, while a rare occurrence in this clinical context, should be considered in at-risk patients especially those presenting with abnormal neurological findings. Prompt recognition may prevent permanent neurological injury and obviate the need for more invasive therapeutic interventions.

## Key points

- Secondary central nervous system lymphoma (SCNSL) is an uncommon presentation in diffuse large B-Cell lymphoma (DLBCL).
- Patients with a CD4 count below 200 and neurological symptoms warrant a gadolinium-enhanced MRI scan.
- Patients with acute urinary attention and weakness warrant an MRI spine to rule out cauda equina syndrome (CES).
- R-CHOP chemotherapy and high dose methotrexate have been shown to have mortality benefit with SCNSL.

## Background

Human immunodeficiency virus (HIV) patients are at increased risk of multiple complications including central nervous system lymphoma (CNSL), an aggressive form of non-Hodgkin’s lymphoma (NHL) [1]. CNSL most commonly affects the brain parenchyma, spine, leptomeninges and eyes [2, 3] but rarely affects the spinal cord. CNSL has a poor prognosis with a median overall survival under 3 years, with worse prognosis for elderly [2]. We present the case of an adult female with HIV, who developed CES in the context of CNSL.

## Case presentation

**Fig. 1:**
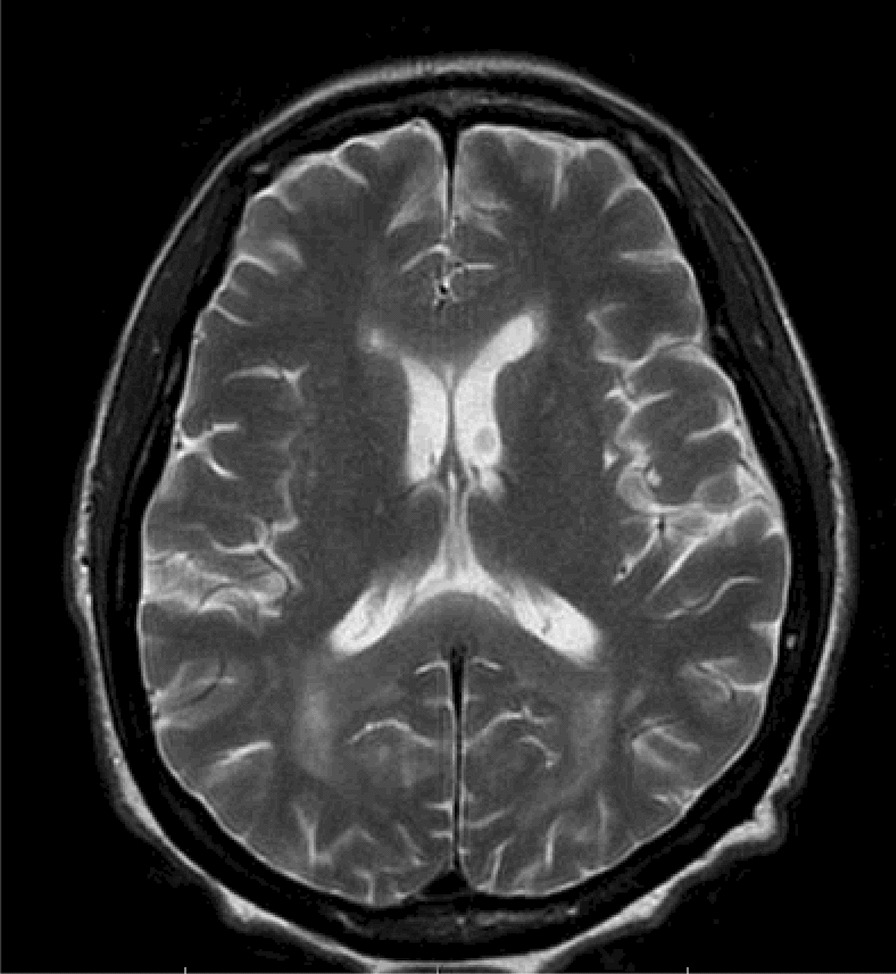
MRI brain with contrast showing significant confluent hyper signal T2 FLAIR within the subcortical and deep ventricular white matter of both cerebral hemispheres, as well as in the corpus callosum, right posterior limb of the internal capsule and cerebral peduncle

**Fig. 2:**
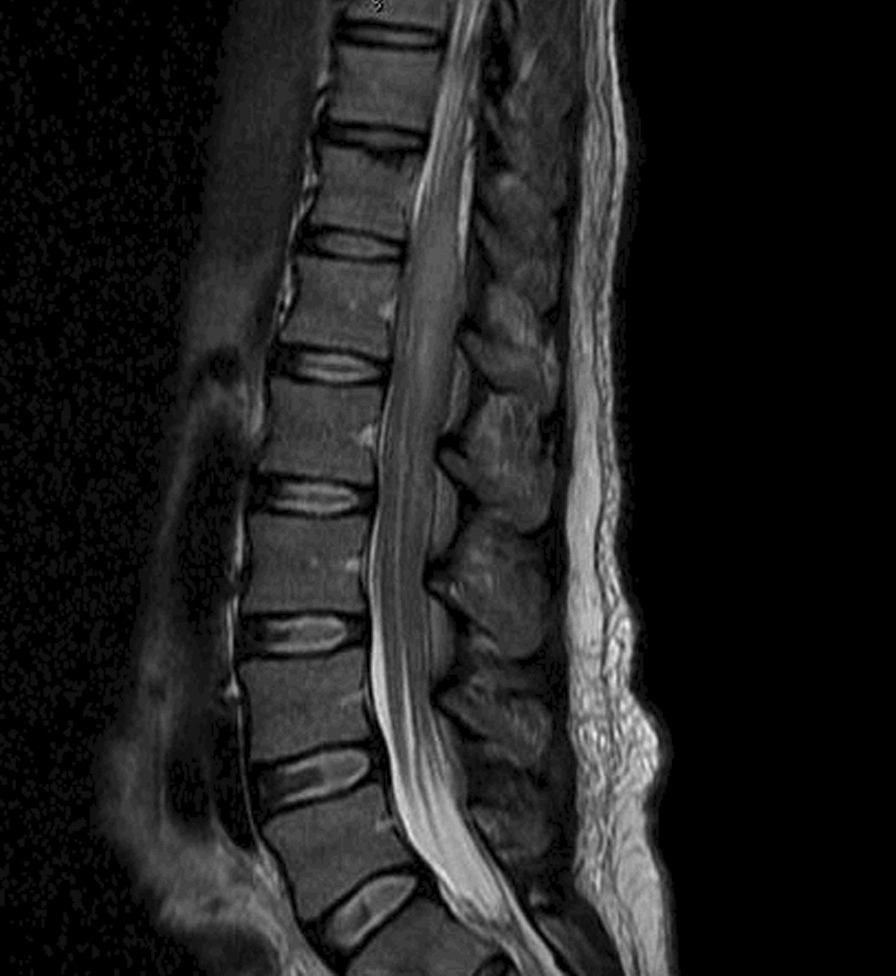
MRI Lumbar-Thoracic Spine with contrast showing large lumbar intradural enhancing mass involving diffusely the nerve roots of cauda equina and conis medullaris. The edema extends from T10 to the conus medullaris. The tumoral component extends to the lumbar neural foramina to the dorsal ganglia

A 46-years old female presented to the emergency room (ER) complaining of bilateral, painful inguinal lymph nodes. She also endorsed progressive fatigue, fever, anorexia, and unintentional weight loss (twenty kilograms in the past month). Her past medical history was significant for Hashimoto’s hypothyroidism (diagnosed in 2010) and HIV (diagnosed in 2011). The patient was unaware she had HIV and was likely diagnosed in her home country of Cameroon. She immigrated to Canada in 2015, and had a medical assessment in 2016 with documentation indicating she had been diagnosed with HIV since 2011. Since at least 2015, the patient had not been on any antiretroviral therapy. To our knowledge, the patient did not have any AIDS defining illness. In terms of her obstetrical history, she was G2P2 and both pregnancies were spontaneous vaginal deliveries. There were no issues with the pregnancies. In Canada, she received social assistance benefits from the government for her living expenses. The patient was not employed but was enrolled in a social program to learn English and French, so that she could participate in the work force. She lived in an apartment with her two children (8 years old male, and 10 years old female). She had no extended family in Canada. Her home medications were levothyroxine (100 mcg PO daily), vitamin D (1000 IU PO daily) and calcium carbonate (500 mg PO BID); she was not on any antiretroviral therapy. The patient denied any smoking, drinking alcohol or using recreational drugs. CT abdomen-pelvis demonstrated enlarged inguinal and intra-abdominal lymph nodes. She was initially discharged from the ER to be seen in hematology clinic however she returned within a few weeks owing to visual and auditory hallucinations. Psychiatric assessment concluded there was an underlying organic cause for her presentation. On assessment, she was alert and oriented to person, place, and time. Her BP was 122/80, her HR 106 regular, her temperature was 36.7, and her oxygen saturation was 96% in room air. Abdominal exam demonstrated significant lymphadenopathy in the inguinal region. Neurological exam demonstrated pyramidal weakness in the right upper extremity as well as both lower extremities. She was hyperreflexive in both triceps and knee reflexes. She had reduced vibration sense in both her halluxes. Labs revealed a normocytic anemia (Hb 107 g/l MCV 90 fL, WBC 5.50 × 10^9^/L, Plt 380 × 10^9^/L) and a mild hypercalcemia (ionized Ca 1.48 mmol/l). LDH was 129 units/liter. Her peripheral blood smear demonstrated a few elliptocytes, rouleaux and polychromasia. It did not demonstrate any blasts. The HIV viral load was 123/mL & CD4 count was 89. She was COVID + but asymptomatic. Her TSH was 3.86 with a normal Free T4. Viral hepatitis panel was negative. Epstein bar virus (EBV) was positive with 6670 copies. Toxoplasmosis IgG was positive with but IgM was negative. Cytomegalovirus (CMV) IgG was positive. Syphilis, cryptococcus and strongyloides serologies were negative. Peripheral blood smear was negative for malaria. CT head, performed prior to lumbar puncture, was unremarkable. Cerebrospinal fluid (CSF) showed 39% lymphocytes and 16% blasts—the report did not state what type of blasts were found therefore the clinical significance of this finding was unclear. CSF cytology was negative for malignancy but showed rare monocytes. Flow cytometry was not performed on CSF. CSF glucose was low [0.4 mmol/L] and protein was high [0.46 g/l]. CSF fluid was negative for tuberculosis, herpes simplex virus (HSV-1 + 2), syphilis, and human polyomavirus two. No bone marrow biopsy was performed. CT chest demonstrated a right upper nodule in the chest with a ground glass appearance, but no thoracic lymphadenopathy. MRI brain with contrast showed significant confluent hyper signal T2 FLAIR within the subcortical and deep ventricular white matter of both cerebral hemispheres, corpus callosum, right posterior limb of the internal capsule and cerebral peduncle (Fig. 1). There was also cranial nerve enhancement. Collectively, these findings were suspicious for leptomeningeal dissemination versus CNS infection. As such, the patient was initially started on ceftriaxone, ampicillin, vancomycin and acyclovir but the antibiotics were soon discontinued after further investigations. The patient was also started on nirmatrelvir/ritonavir (Paxlovid) for COVID. She was also started on bictegravir, emtricitabine, and tenofovir (Biktarvy). Despite a week of anti-retroviral therapy (ART) her CD4 count dropped to 49. A left inguinal node biopsy with flow cytometry showed diffuse large B-cell lymphoma (DLBCL). Flow cytometry also showed BL6, myc rearrangement (double-hit rearrangement), and CD20 positive. During her admission, the patient developed new urinary retention and new worsening bilateral lower limb weakness, resulting in the inability to walk. MRI lumbar-thoracic spine demonstrated a large lumbar intradural enhancing mass diffusely involving the nerve roots of the cauda equina and conus medullaris (Fig. 2). Based on these findings the patient was diagnosed with SCNSL with intramedullary dissemination causing CES. The patient had Stage IV DLBCL. The patient was urgently transferred to another center to initiate high dose methotrexate (3 g/m^2^) and induction chemotherapy: cyclophosphamide, doxorubicin, vincristine, prednisone and rituximab (R-CHOP). The use of high dose methotrexate was justified based on previous studies which have used similar regiments to manage this complication. We did not repeat the LP to analyze the CSF. The patient also developed a subsegmental right lower lobe pulmonary embolism, found on a CT pulmonary angiogram. She was started on therapeutic low molecular weight heparin (LMWH) for 6 months. Following therapy, our patient regained the ability to walk and recovered neurological function. A repeat whole body PET scan demonstrated a reduction of hypermetabolic foci throughout the body post treatment. MRI of the cervical spine still showed suspected leptomeningeal enhancement in the anterior cervical spinal cord of C1/C2. There were no adverse or unanticipated events during the patient’s treatment.

## Discussion

In summary, this is a 46 years old female that presented with hallucinations and eventually developed cauda equina syndrome from DLBCL on the background of untreated HIV. This is case is very unique in that it is quite rare for a SCNSL to cause CES on the background of untreated HIV.

Life expectancies of HIV infected individuals on ART have now equilibrated with their non-infected counterparts. However, with inadequate treatment/treatment resistance, 25–40% may develop a malignancy, with 10% of these being non-Hodgkin lymphoma (NHL) [4] Key risk factors for NHL in HIV infected individuals include a CD4 count below 100 and HIV RNA viral levels exceeding 1000 copies [5]. In cases of CNS lymphoma, the CD4 count is often lower than 50 [6] as in the current case. There are three main categories of HIV associated NHL: systemic NHL, primary CNS NHL, and primary effusion NHL. DLBCL accounts for 75% of NHL [7] with 2–10% at risk of CNS dissemination [8]. The symptoms of SCNSL occur acutely over days to weeks. Furtheremore, in the case of our patient, there may be leptomeningeal dissemination (LM) occurring in up to 8% of patients [9]. LM often manifests with cranial nerve deficits, radicular pain, change in mental status, focal weakness, sensory loss, or headache. Our patient initially presented with severe change in her mental status and weakness, which resolved after treatment. In addition, spinal cord dissemination or intramedullary dissemination (IMD) may occur when there is a tumour in the subarachnoid space growing along the nerve roots or direct hematogenous tumour spread. IMD is a rare occurence and therefore the prevalence cannot be reliably estimated. The typical presentation includes weakness, bowel and bladder dysfunction, spasticity, pain, and sensory loss. Our patient experienced urinary retention and weakness secondary to CES as a result of IMD. Other sequelae of SCNSL include the presence of parenchymal brain metastases. Initially it was thought LM was more common that parenchymal involvement, however a recent retrospective analysis demonstrated that among patient’s with CNS involvement, 43% had parenchymal involvement, while 40% had LM [10]. Symptomology depends upon where the metastases are located. Another sequelae is neurolymphomatosis which involves direct involvement of peripheral nerve roots or cranial nerves. This is an uncommon phenomenon and its identification requires multiple imaging techniques, nerve biopsy and a high degree of suspicion. In our patient, neither of these sequelae developed however they should be considered in patients with suspected SCNSL.

There is no set criteria for diagnosing SCNSL, however diagnosis generally requires radiographic imaging of the brain and the spine with positive CSF cytology. The imaging modality of choice is a gadolinium-enchanced MRI. The diagnosis of SCNSL may also be confimed with biopsy of the involved nerve roots or of brain metastasis. In the case of our patient, flow cytometry of an inguinal lymph node biopsy was adequate to confirm the presence of DLBCL and imaging findings demonstrated CNS involvement. Our patient’s lymph node biopsy demonstrated *BLC-6* positive and a *myc* rearrangement. The patient was also found to be EBV positive. Almost all HIV SCNSL have an EBV coinfection [11]. It is theorized that EBV and other coinfections permit the proliferation of deranged B cells that have undergone changes in their oncogenes or tumour suppression genes, especially in the *c-myc* oncogene [12].

**Fig. 3:**
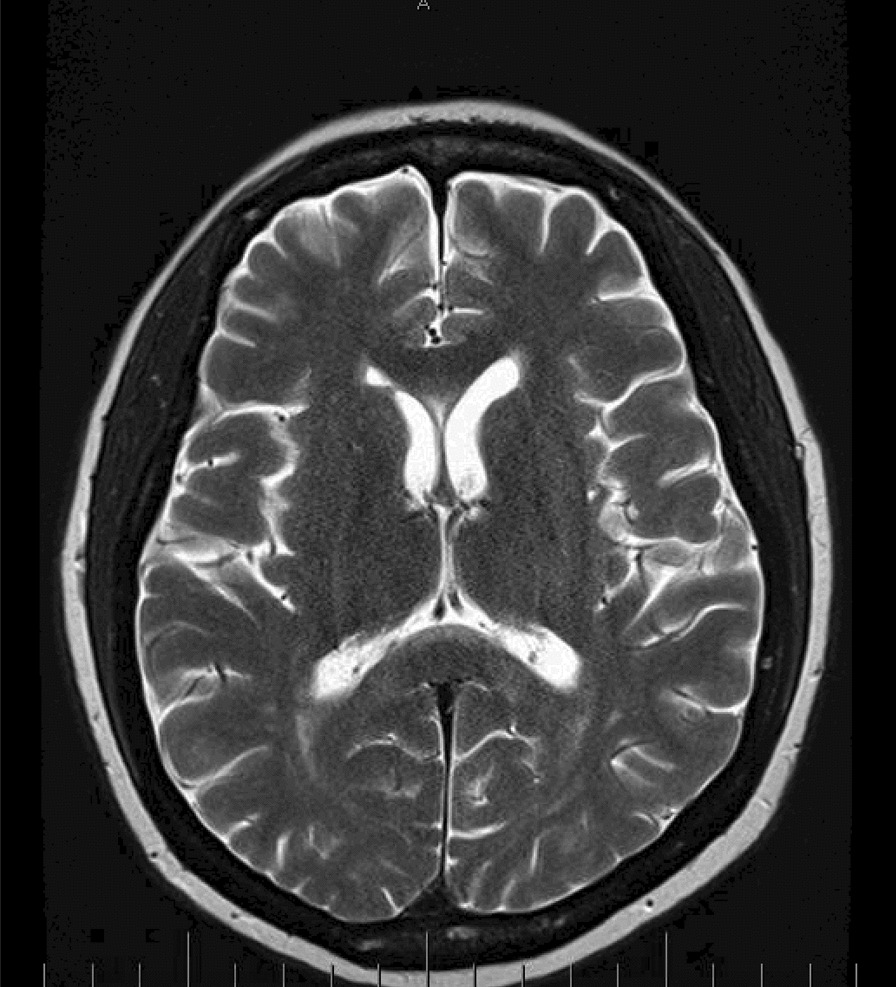
The gadolinium enhancement has changed, with resolution of the previously seen enhancement along the right 7th and 8th nerves, resolution of the previously seen enhancement along the optic chiasm, but new enhancement within the perivascular spaces penetrating through the cerebral hemispheric white matter. This would indicate the presence of leptomeningeal enhancement. The white matter disease although still present, has become slightly less severe, but still consistent with a diffuse leukoencephalopathy pattern

The mainstay of treatment for SCNSL is R-CHOP with high dose methotrexate (MTX), because this regimen penetrates the blood brain barrier and has demonstrated efficacy in promoting survival in this population. Patients with refractory or relapsed HIV lymphoma may consider autologous hematogenous stem cell transplant (HCT). Our patient was started on standard DLBCL chemotherapy protocol: R-CHOP, high dose methotrexate (3 g/m^2^) therapy and ART. High dose methotrexate combined with ART has been shown to increase long term survival of AIDS related CNSL and reduce relapses [13]. The patient’s viral load decreased from 123/mL to less than 20, and her CD4 count increased from 89 to 152. Our patient responded well to this regimen both clinically and radiographically (Fig. 3). She had received 6 cycles of R-CHOP and 6 cycles of high dose MTX.

SCNSL confers a poor prognosis a median survival of 2.2 months after diagnosis [8]. However, previous studies have not examined outcomes with newer treatments such as rituximab or high dose MTX with autologous HCT. Therefore, it is likely that prognosis may in-fact be better than previously thought. Our patient has survived greater than 6 months since diagnosis, and is responding well to treatment both clinically and radiographically.

The strength of this case report is that we highlight a rare instance of SCNSL causing cauda equina syndrome. We also provide radiographical evidence to support this claim and evidence of clinical response to therapy. Limitations of this case report include the fact that this is a single example of this occurrence with limited generalizability to other patient populations as this patient had been previously non-compliant with medication. As such, in patients who are compliant, this occurrence may be less likely and therefore the findings of this case report less relevant. Notwithstanding, given the significant number of patients with uncontrolled HIV, the findings of this case report are especially relevant to this at-risk population.

The patient was followed by hematology as an outpatient. Once the patient was initiated on R-CHOP and high dose methotrexate and anti-viral therapy, her lymphoma improved. Subsequent PET-CT and MRI scans showed decrease in the avidity.

## Conclusion

In conclusion, patients with HIV and focal neurological deficits, should undergo an urgent MRI of the CNS given the risk of SCNSL in these individuals. Prompt recognition may prevent permanent neurological damage.

## Data Availability

All data generated or analyzed during this study are included in this article. Further enquiries can be directed to the corresponding author.

## Abbreviations

HIV: Human immunodeficiency virus
CNSL: Central nervous system lymphoma
NHL: Non-Hodgkin’s lymphoma
CSF: Cerebrospinal fluid
ART: Anti-retroviral therapy
DLBCL: Diffuse large B-cell lymphoma
LM: Leptomeningeal dissemination
IMD: Intramedullary dissemination
R-CHOP: Cyclophosphamide, doxorubicin, vincristine, prednisone and rituximab
